# Structural Health Monitoring of a Composite Panel Based on PZT Sensors and a Transfer Impedance Framework

**DOI:** 10.3390/s18051521

**Published:** 2018-05-11

**Authors:** Michal Dziendzikowski, Patryk Niedbala, Artur Kurnyta, Kamil Kowalczyk, Krzysztof Dragan

**Affiliations:** Air Force Institute of Technology, ul. Ks, Boleslawa 6, 01-494 Warszawa, Poland; patryk.niedbala@itwl.pl (P.N.); artur.kurnyta@itwl.pl (A.K.); kamil.kowalczyk@itwl.pl (K.K.); krzysztof.dragan@itwl.pl (K.D.)

**Keywords:** Structural Health Monitoring, PZT transducers, composite structures monitoring, damage detection

## Abstract

One of the ideas for development of Structural Health Monitoring (SHM) systems is based on excitation of elastic waves by a network of PZT piezoelectric transducers integrated with the structure. In the paper, a variant of the so-called Transfer Impedance (TI) approach to SHM is followed. Signal characteristics, called the Damage Indices (DIs), were proposed for data presentation and analysis. The idea underlying the definition of DIs was to maintain most of the information carried by the voltage induced on PZT sensors by elastic waves. In particular, the DIs proposed in the paper should be sensitive to all types of damage which can influence the amplitude or the phase of the voltage induced on the sensor. Properties of the proposed DIs were investigated experimentally using a GFRP composite panel equipped with PZT networks attached to its surface and embedded into its internal structure. Repeatability and stability of DI indications under controlled conditions were verified in tests. Also, some performance indicators for surface-attached and structure-embedded sensors were obtained. The DIs’ behavior was dependent mostly on the presence of a simulated damage in the structure. Anisotropy of mechanical properties of the specimen, geometrical properties of PZT network as well as, to some extent, the technology of sensor integration with the structure were irrelevant for damage indication. This property enables the method to be used for damage detection and classification.

## 1. Introduction

Application of elastic waves is one of the most universal approach to non-destructive testing of materials [1]. For excitation and detection of elastic waves, materials which exhibit piezoelectric properties need to be used [2]. Commonly applied as elastic waves actuators and sensors are transducers based on lead-zirconate-titanate (PZT) ceramics, i.e., PZT transducers [3,4]. Also, for over two decades now, PZT transducers have been applied to Structural Health Monitoring [5,6,7]. Elastic waves can interact with damages of different types [8], also they can propagate over long distances from their source, e.g., a PZT actuator. Therefore, sparse networks of PZT transducers can be used for monitoring relatively large structures, independently of the material used for manufacturing the structure. Due to those properties, PZT transducers have found a variety of applications in SHM. They can be applied to:

- fatigue crack and corrosion detection [9,10,11,12,13,14];
- bolt and bolted joint monitoring [15,16];
- adhesive and welded joint monitoring [17,18,19,20,21];
- detection of impact damage of composite materials [22,23,24];
- civil structure monitoring [17,18,19,20,21,22,23,24,25,26];
- transducers’ self-diagnosis [26,27,28,29,30,31,32,33,34,35,36,37].

The application of PZT sensors to SHM is always based on the propagation of elastic waves, usually Lamb waves, through a monitored structure [5,6,7,8]. However, four different setups of PZT transducers can be distinguished in applications. There are two factors allowing for distinction between the setups [5,6,38,39]:

- the configuration of the PZT transducers;
- the way how the PZT transducer used for elastic wave excitation, i.e., the PZT actuator, is sourced with voltage.

**Figure 1:**
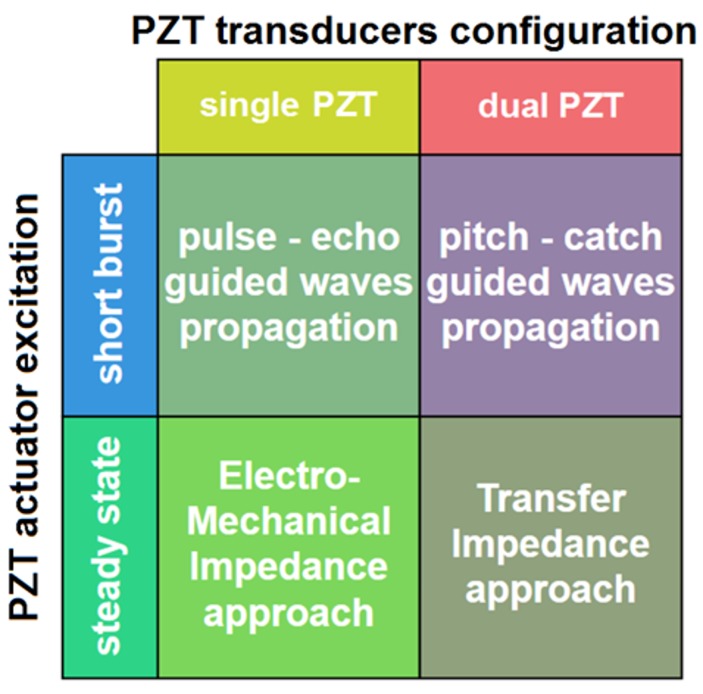
Methods of PZT application for Structural Health Monitoring.

The terminology of this distinction adopted for the purpose of this article is presented in Figure 1.

The first factor which can be considered when using PZT transducers for SHM purposes is their configuration. First, a single PZT transducer can be used for damage detection of structures [6,10,11,20,21]. The transducer has a dual role then—it is a source of elastic waves, i.e., it is a PZT actuator, but it is also a receiver of waves scattered and reflected from structure discontinuities surrounding the transducer, i.e., it is a PZT sensor. Waves reflected from a damage of a finite size decay with the distance from it [4], therefore whether they can be detected by a PZT transducer depends on the distance of the damage from it. Also, pairs of PZT transducers can be used for structure monitoring—one of the transducers is exciting elastic waves, i.e., it is a PZT actuator, whereas the second one is receiving them, i.e., it is a PZT sensor [5,12,13,15,16,17,18,38]. If pairs of PZT transducers are used, the so called transmission mode of elastic wave interaction with a damage can be utilized in addition to the reflection mode [8]. Elastic waves propagating between PZT actuator and PZT sensor can be distorted during their transmission through the encountered damage. Due to this phenomena, the signal acquired by the PZT sensor can be changed. Signal changes should occur irrespective of the distance between the PZT actuator and the PZT sensor, as long as damage is big enough to cause significant transmission effects.

According to the scheme presented in Figure 1, the second factor which defines the approach to SHM is the type of PZT actuator excitation. The actuator can be sourced with a short pulsed or steady state sinusoidal voltage. In the first case [5,13,14,15,16,23,24,38], the voltage on the PZT sensor is acquired over a certain period. For the pulse-echo wave propagation approach, i.e., a single PZT transducer excited with a short pulsed voltage, the acquired signal is due to waves reflected from structure discontinuities surrounding the transducer. For the pitch-catch wave propagation approach, i.e., if a PZT actuator—PZT sensor pair is used, the voltage on the sensor is induced by waves transmitted directly between PZT transducers as well as waves reflected from structure discontinuities located within the sensing range of the transducers. If a PZT actuator is sourced with a steady state sinusoidal voltage, then the current flow or the induced voltage on PZT sensor are used for structure assessment. Both type of response signals measured on a PZT sensor are of sinusoidal type and have the same frequency as the voltage applied to the PZT actuator. The amplitude ratio and the phase shift between the response signal and the voltage applied to the PZT actuator can be can changed due to interaction of excited elastic waves with a damage, therefore they can be useful for damage detection. The frequency sweep of the PZT actuator excitation is used, in order to obtain response signal characteristics in a broad bandwidth since different type of damage can influence the signal for different frequency range. For the Electro-Mechanical Impedance approach [6,10,11,20,21,22], i.e., when PZT transducer is both the actuator and the sensor, only the current flow can be used as the response signal. For the Transfer Impedance approach [17,40,41,42,43] both types of response signals acquired by the PZT sensor are eligible to be used for structure assessment.

In this article a method of structure monitoring based on the Transfer Impedance (TI) approach is presented. For the purpose of structure assessment, a set of Damage Indices (DIs) is proposed, based on the voltage induced on PZT sensor. The proposed DIs maintain the same information about the structure as the response signal, therefore all of the damage detection capabilities of the TI approach should be preserved by the proposed method. The properties of the proposed DIs were investigated experimentally. In particular, repeatability of damage indication for an anisotropic medium was verified—a GFRP composite panel was used as a test specimen in the experiment. Also, comparison of the methods performance for PZT transducers embedded into the composite structure and attached to the specimen surface is presented in the article.

The rest of the article is organized as follows: in the next section the description of the proposed method for structure monitoring is provided. It is followed by a detailed description of the experimental design, including the equipment used for the study. Then, the main findings of the research and conclusions are presented.

## 2. Materials and Methods

**Figure 2:**
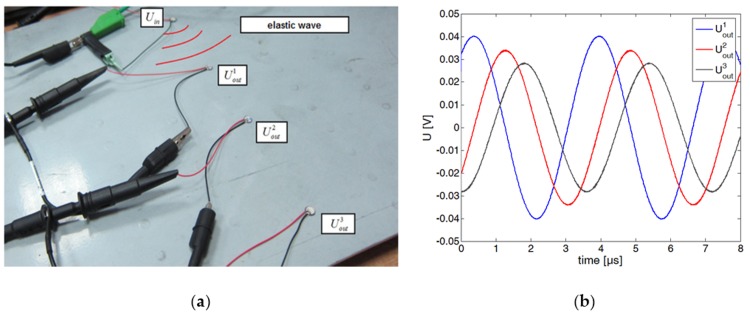
Example of a sinusoidal steady state excitation of PZT sensors: (**a**) PZT network; (**b**) Output voltages acquired on PZT sensors.

As mentioned in the introduction, one of the methods for PZT transducer application to damage detection is to apply a sinusoidal steady state voltage to a PZT actuator and measure the voltage on a PZT sensor, which is induced by elastic waves propagating between the PZT actuator—PZT sensor pair. In Figure 2, an example of voltage signals $U_{out}^{i}$, i=1,2,3 are presented, which were obtained for three different PZT sensors receiving elastic waves excited by an actuator sourced with sinusoidal voltage $U_{in}$. If a sinusoidal voltage—$U_{in}$ is applied to PZT actuator, then the induced voltage on the sensor—$U_{out}$ is also sinusoidal and has the same frequency as $U_{in}$. Thus the ratio:

(1) $$TF=\frac{U_{out}}{U_{in}},$$

called the transfer function [17,41], does not depend on time and it can be written in complex form as:

(2) $$TF(\omega )=\frac{U_{out}}{U_{in}}=\frac{|U_{out}|e^{i\omega t+\varphi (\omega )}}{|U_{in}|e^{i\omega t}}=|TF(\omega )|e^{i\varphi (\omega )},$$

where |TF(ω)|, φ (ω) denote respectively—the amplitude ratio and the phase difference between output $U_{out}$ and input $U_{in}$ signals for a given frequency ω. Equivalently, the transfer function can be written using its real and imaginary part as:

(3) TF(ω)=ReTF(ω)+iImTF(ω),

where:

(4) ReTF(ω)=|TF(ω)|cosφ(ω),  ImTF(ω)=|TF(ω)|sinφ(ω)

**Figure 3:**
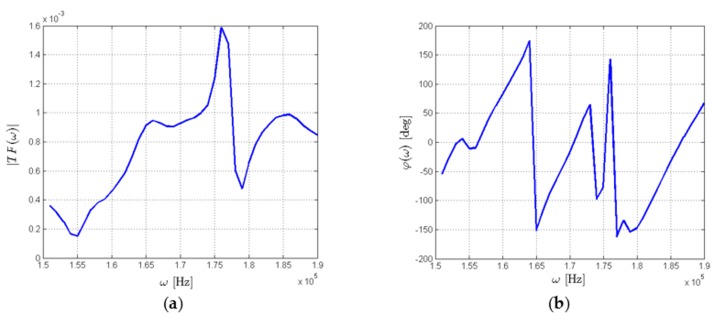
Example of transfer function components: (**a**) amplitude ratio |TF(ω)|; (**b**) phase difference φ (ω), between output and input signals.

This transfer function is to a large extent invariant with respect to disturbances of the excitation voltage $U_{in}$, which is one of its advantages. However, its direct use is of limited usability for structure assessment due to complex dependence of TF(ω) on the frequency of the excitation signal ω. As an example, components of transmission function obtained for a simple structure are presented in Figure 3. 

Both components of TF(ω)—its amplitude and the phase—carry the information about mechanical properties of the structure within the sensing range of a given pair of transducers, but also depend on other factors, e.g., the properties of the PZT transducers used or the distance between them. Taken together, this results in a nontrivial behavior of the transfer function with respect to the frequency of the voltage $U_{in}$ applied to the PZT actuator.

Therefore, the state of the structure is usually assessed using some comparative signal characteristic, called the Damage Index (DI). DIs measure the difference between transfer function TF(ω) obtained for the actual state of the structure and the baseline transfer function $TF_{0}\left(\omega \right)$, obtained for the initial state of the structure. Usually, DIs are based on real or imaginary part of transmission functions, e.g., they can be written as [5,6,19,37]:

(5) DIRMSD=∫Ω(ReTF(ω)−ReTF0(ω))2dω∫Ω(ReTF0(ω))2dω,  DIcor=1−corr(ReTF,ReTF0),

where Ω is the bandwidth of the excitation signal and $corr\left(ReTF,\;ReTF_{0}\right)$ denotes the correlation coefficient between real parts of the actual and the baseline transmission functions. Such DIs project all of the information contained in the transmission function to a single value. DI values should represent the scale of difference between transmission functions obtained for the two states of the structure. If there is no change in the signals, values of DIs are usually supposed to be close to zero, whereas if damage is present, their values should be significantly different than zero.

In this paper, it is proposed to intertwine real and imaginary parts of transmission function and to consider the complex quotient as DIs:

(6) $$DI(\omega )=\frac{TF(\omega )}{TF_{0}(\omega )}=\frac{\left|TF(\omega )\right|}{\left|TF_{0}(\omega )\right|}e^{i(\varphi (\omega )-\varphi_{0}(\omega ))},$$

where $\left|TF_{0}\right|$ and $\varphi_{0}$ denote the components of the baseline transfer function $TF_{0}\left(\omega \right)$. Equivalently, the proposed DIs can be written as:

(7) DI(ω)=ReDI(ω)+iImDI(ω),

where:

(8) ReDI(ω)=|TF(ω)||TF0(ω)|cos(φ(ω)−φ0(ω)),  ImDI(ω)=|TF(ω)||TF0(ω)|sin(φ(ω)−φ0(ω)).

Assuming, that the voltage on the generator $\;U_{in}$ is the same for TF(ω), $TF_{0}\left(\omega \right)$, the modulus of DIs satisfies the following relation:

(9) $$|DI(\omega )|=\frac{TF(\omega )}{TF_{0}(\omega )}=\frac{\left|U_{out}(\omega )\right|}{\left|U_{0,out}^{}(\omega )\right|},$$

therefore, it describes the ratio between voltage amplitudes induced by elastic waves on the sensor.

**Figure 4:**
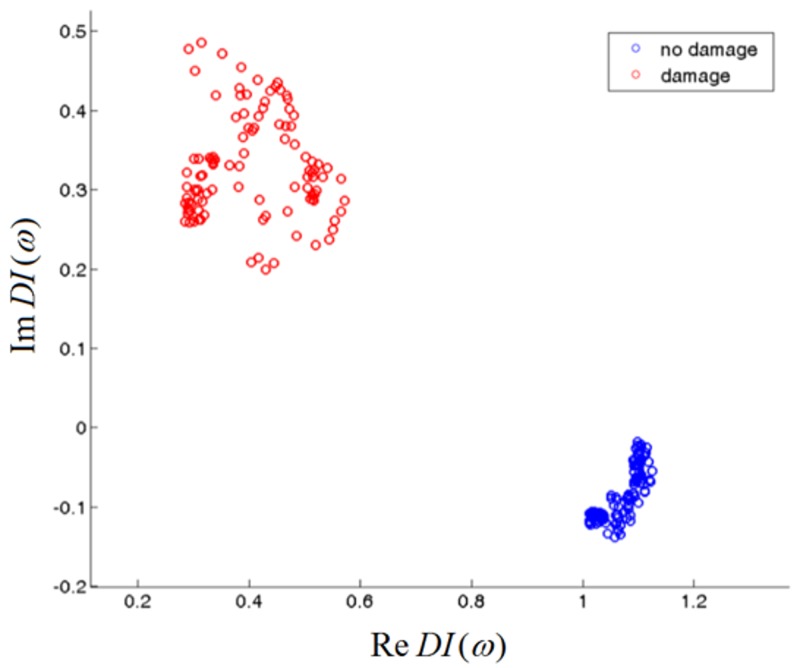
Example of DIs obtained for the pristine state of the structure and when a damage is present.

The proposed DIs utilize both components of transmission functions and captures all of the information about the output voltage amplitude and its phase changes. Thus, if a damage of a given type impacts the amplitude of the output voltage or cause its phase shift, it should be captured by the proposed DIs. The type of damage which are detectable by Electro-Mechanical Impedance or Transfer Impedance methods are listed in the introduction. For structure assessment, the DIs obtained for frequencies ω belonging to a given bandwidth Ω can be represented in the complex plane. In Figure 4 an example of DIs’ behavior obtained for undamaged structure and under damage presence is illustrated. For undamaged structure, the DIs should be concentrated in the vicinity of the point 1 + *i*0 in the complex plane, irrespectively of the frequency of the excitation. If damage is present, it can change the output voltage amplitude or its phase, therefore DIs should diverge from the point 1 + *i*0 (Figure 4).

In the presented example all of the frequencies were influenced by a damage, therefore a single frequency could be used for structure assessment in this case. However, it is not necessary to limit the data and to use DI value obtained at a given frequency ω for the purpose of damage detection. Rather DIs’ behavior obtained for a range of frequencies should be used as it is better suited for damage detection and classification. It is possible that damage of a certain type would affect the DIs only in a limited frequency range. In such case two groups of data would be formed in the complex plane: the DIs obtained for frequencies not sensitive to damage would be close to the point 1 + *i*0, whereas DIs influenced by damage would be separated from it. In this way optimal range of frequencies for the purpose of damage detection could be established.

Also it is possible that damages of different types could be located at different domains in the complex plane. In this case it would be possible to classify type of damage based on DIs’ behavior using statistical methods of data classification, e.g., nearest neighbor algorithm or linear discriminant classifier [37]. This is hard to achieve using classical DIs, as presented in the Equation (5), since all of the information about the amplitude and the phase changes of the response signal is projected to a single value. A necessary condition for classification methods to work properly, is repeatability of DIs indications. DIs should be located at the same region of the complex plane when comparable damage is present within the sensing range of PZT actuator and PZT sensor. This property of the proposed DIs was verified experimentally for a particular type of damage. The obtained results are presented and discussed further within the text.

## 3. Design and Preparation of the Experiment

This section provides detailed description of the experiment designed to verify basic properties of the Damage Indices proposed in the paper. The outcome of the experiment was assumed to be:

- verification of indications stability for a given sensing path under certain conditions;
- comparison of indications for different sensing paths under certain conditions;
- comparison of method performance for PZT transducers embedded into the composite structure and attached to its surface;

where the sensing path is defined to be a pair: PZT actuator—PZT sensor.

### 3.1. Specimen and PZT Network Description

The specimen used for the experiment was a GFRP panel made of 16 plies of HCS2401-015—HEXCEL Fiberglass Prepreg (Hexcel Corp., Stamford, CT, USA). The stacking sequence of the layers was [0/45/0/45/0/45/0/45]_s_. In the symmetry plane of the specimen, a network of SMD05T04R111 PZT discs made by STEMINC Inc. (Doral, FL, USA) was deployed. The diameter of PZT transducers used for the experiment was 5 mm and their thickness was 0.4 mm. After PZT transducer deployment, the specimen was cured in the autoclave in accordance with technical specification of the material used.

**Figure 5:**
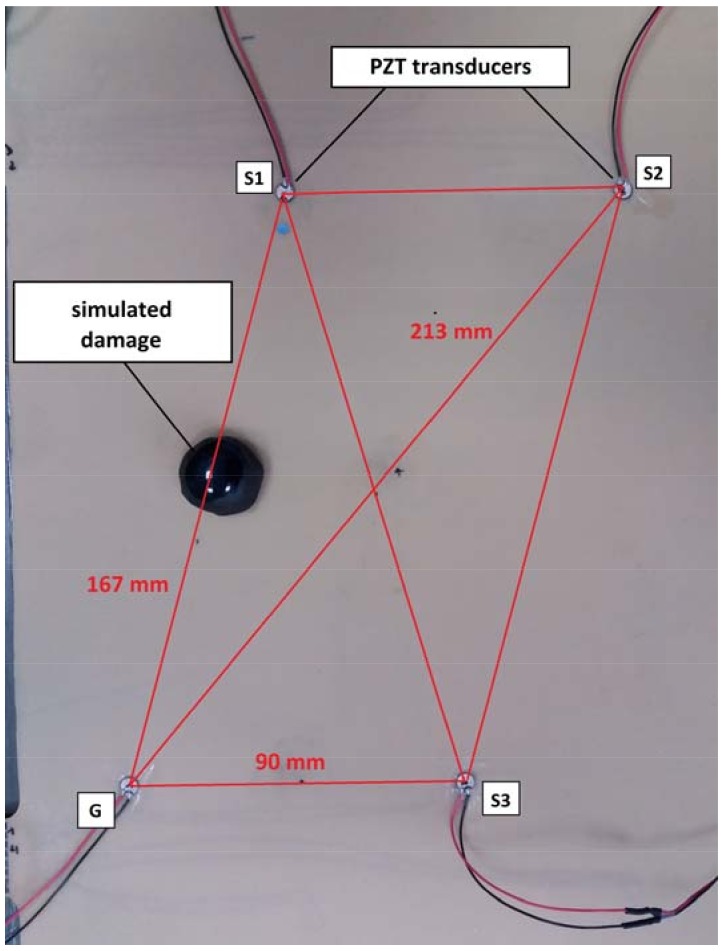
The geometry of the PZT network.

Four PZT transducers of the same type were deployed on the surface of the specimen, precisely above four selected PZT transducers embedded in the specimen structure. The orientation of the attached transducers was maintained the same as for the embedded transducers. In Figure 5 the geometry of the PZT networks is presented. For each network, one of PZT transducers was selected as PZT actuator whereas the remaining three transducers were used as PZT sensors. The positions of the actuator G, and the sensors S1, S2, S3 are indicated in Figure 5. For each network three sensing paths were used (Figure 5):

- G-S1 of the length 167 mm;
- G-S2 of the length 213 mm;
- G-S3 of the length 90 mm.

It is worth noticing that not only the lengths of sensing paths were different in the experiment, but also their orientation with respect to specimens plies and orientation between PZT actuator and PZT sensors. Such configuration of network allows to investigate influence of anisotropy of GFRP specimen and PZT transducers [5] on DIs’ behavior.

### 3.2. Hardware and Software Description

**Figure 6:**
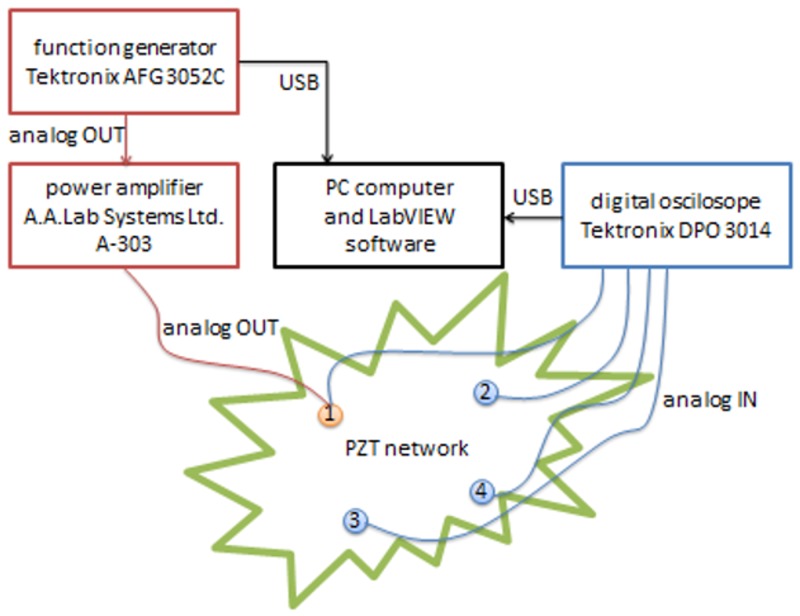
Block diagram of the measurement system.

**Figure 7:**
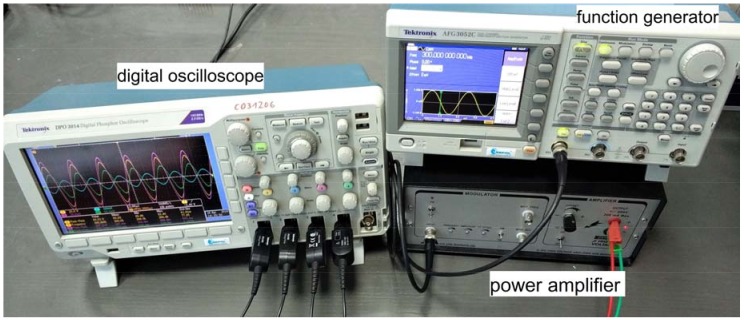
The system devices prepared for measurements.

The measurement setup used in the experiment included a PC computer, digital oscilloscope, arbitrary function generator and power amplifier (Figure 6 and Figure 7).

The function generator was used to provide a sinusoidal signal with constant predefined amplitude in a given frequency range. The power amplifier was used for signal conditioning both for the voltage (20×) and for the current (up to 200 mA) in order to operate PZT actuator in the frequency regime where impedance of the actuator is low, i.e., close to the resonant frequency of piezo elements used [2,18]. The digital oscilloscope was used for measuring the excitation voltage on the PZT actuator—G—as well as induced voltage on the three sensors: S1, S2, S3. The devices were connected to a PC computer with an installed LabVIEW environment.

**Figure 8:**
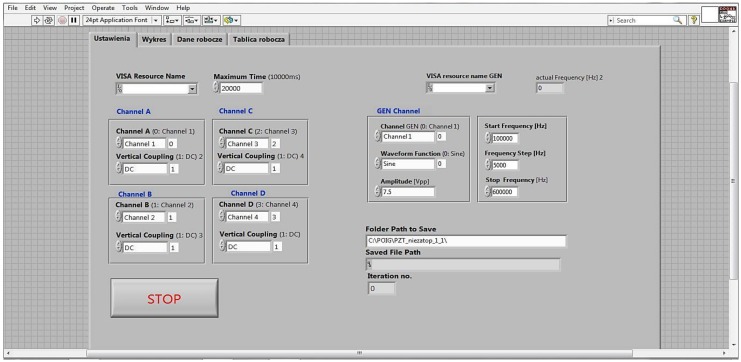
The front panel of the developed computer program—the settings window of the oscilloscope and the function generator parameters.

An application in the LabVIEW environment was developed, which allowed automatization of the measurement process, configuration and synchronization of the devices, as well as visualization and storage of the acquired data. In Figure 8, the setup panel of the application is presented.

**Figure 9:**
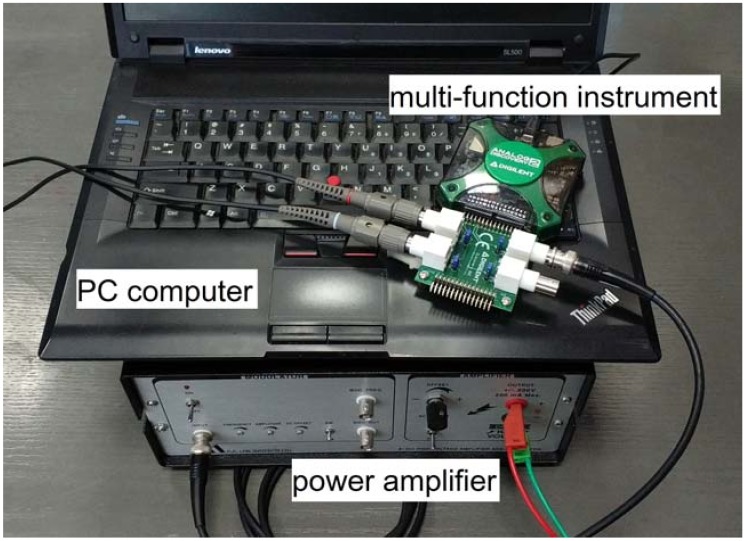
Parts of the integrated measurement system.

The typical laboratory setup (Figure 6 and Figure 7) was used for convenience. Instead, it is possible to use an integrated measuring device like a Digital Analog Discovery 2 that combines a dual channel oscilloscope and generator functions into one device (Figure 9). It can be connected to a portable computer and controlled from the same LabVIEW application as the main system (Figure 8).

In such a solution the presence of a power amplifier is still required and since fewer input channels are available, an additional module switching active PZT sensor pairs based on external mux module would be also necessary. In addition, this kind of devices allows lower measurement accuracy and poorer configuration capability, but, nevertheless, it significantly limits the dimensions of the measurement system and makes it more portable. Therefore, it improves the applicability of the method in real applications.

### 3.3. Measurements Scheme

The measurements were performed in the following order:

(1) Measurement for the pristine state of the structure.
(2) Measurement for simulated damage on sensing path G-S1.
(3) Measurement for simulated damage on sensing path G-S2.
(4) Measurement for simulated damage on sensing path G-S3.

The above measurement scheme was repeated six times, thus data from 24 measurements series were obtained for each network.

The damage was simulated by a mass element attached to the specimen in the middle of a given sensing path (Figure 5). The mass diameter was 22 mm. The mass was attached by a bituminous substrate. The total weight of the mass and the substrate was approximately 6.4 g. The mass provided local pressure field which can distort elastic waves propagating underneath, e.g., the speed of wave propagation can be changed locally [5,8]. In addition, the substrate used for the mass attachment can attenuate elastic waves. Therefore both the amplitude and the phase changes of the response signal were expected. The specimen was not permanently damaged, therefore it was possible to simulate similar condition in the subsequent series of measurements. It allowed for the verification of repeatability of DIs indications.

The actuator G was powered with a sinusoidal steady state voltage in the frequency range of 240–350 kHz. The frequency increment step was 1 kHz and peak-to-peak amplitude of the excitation was set to 88 V. The frequency range was selected based on impedance characteristics of PZT sensors used for the study [23]. Usually, PZT transducers have very high impedance for low frequencies, which then decreases to zero when the frequency goes to the so-called resonant frequency. The lower bound of the frequency band was chosen in order to obtain at least 20 mV peak-to-peak voltage amplitude on the sensors S1, S2, S3. This level of signal allowed for proper calculation of the transfer function components, especially the phase difference φ(ω) between voltage induced on PZT sensor and voltage applied to PZT actuator. The upper bound of the frequency interval was selected to be below the current efficiency of the amplifier as well as the resonant frequency of PZT actuator. This allowed to avoid non-linear effects which may occur in this frequency regime. Therefore, the frequency range used in the experiment was as broad as possible, considering limitations of the devices used for the study.

**Figure 10:**
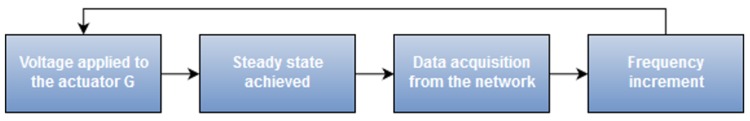
Data acquisition scheme.

For a given measurements series, the data was collected according to the scheme shown in Figure 10:

Damage Indices for each series of measurements were calculated in accordance with the Equation (6). The first measurement obtained for the pristine state of the structure was selected as the baseline transmission function $TF_{0}$. For each series of measurements, the two-dimensional median $DI_{50}$ of the DIs was determined, i.e., the median for real and imaginary parts of DIs. In order to avoid outlying observations only 80% of data closest to the point $DI_{50}$ in the complex plane were considered as valid.

## 4. Results and Discussion

**Figure 11:**
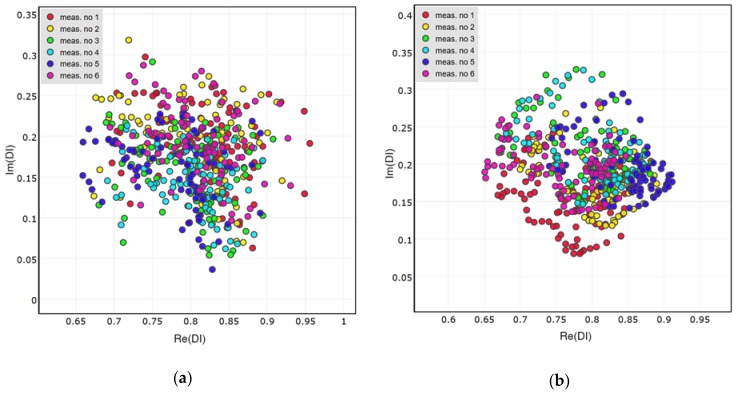
Damage Indices obtained for simulated damage on sensing path G-S1: (**a**) Attached network; (**b**) Embedded network.

**Figure 12:**
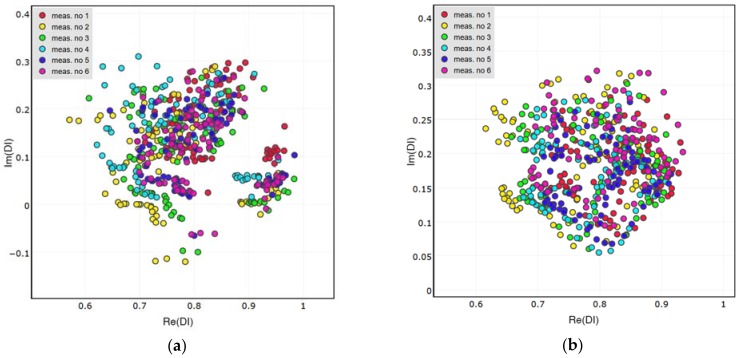
Damage Indices obtained for simulated damage on sensing path G-S2: (**a**) Attached network; (**b**) Embedded network.

**Figure 13:**
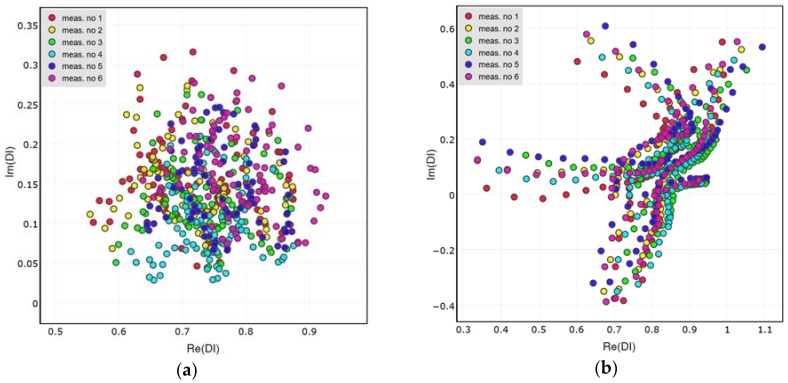
Damage Indices obtained for simulated damage on sensing path G-S3: (**a**) Attached network; (**b**) Embedded network.

**Figure 14:**
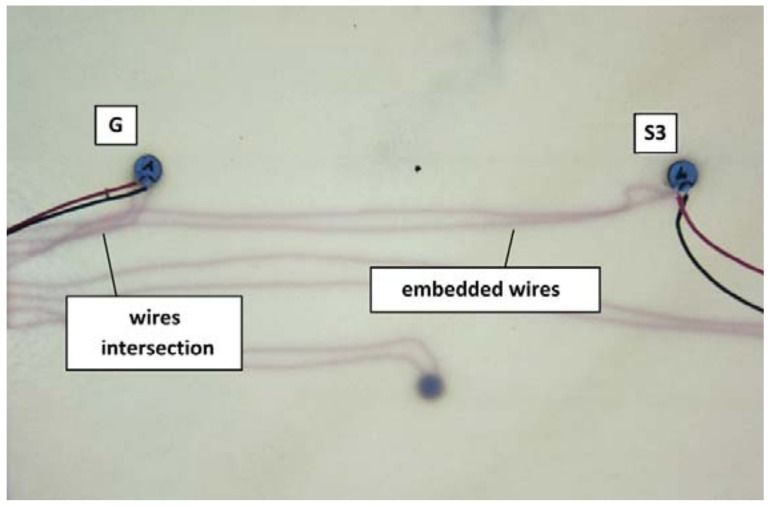
Embedded wires intersection.

Distributions of DIs obtained for the presence of simulated damage are presented in Figure 11, Figure 12 and Figure 13 below. Both the attached and the embedded PZT networks are considered. DIs obtained for repeated measurement condition, i.e., presence of simulated damage, are concentrated around a similar point in the complex plane, therefore repeatability of the proposed method was confirmed. As expected, both the amplitude and the phase of voltage induced on PZT sensors are affected by simulated damage. Also, similar pattern of DIs distribution can be observed for different sensing paths, except for the embedded sensing path G-S3 (Figure 13b). In this case the spread of data is significantly higher compared to other cases. This can be caused by improper configuration of the embedded wires connected to G and S3 embedded transducers (Figure 14). The wires connected to actuator G, to which high voltage was applied, runs in parallel to wires connected to the sensor S3, used for low voltage measurement. This could have impact on the voltage induced on the sensor S3 and interfere with the results, resulting in increased spread of data in that case.

**Figure 15:**
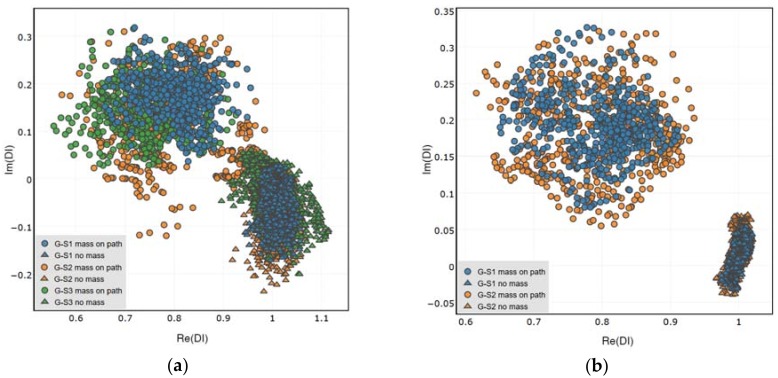
Comparison of DIs obtained for the pristine state of the structure and for simulated damage presence: (**a**) Attached network; (**b**) Embedded network.

In Figure 15, a comparison of the DIs’ distribution for different sensing paths is presented. In the comparison, both states of the structure are considered—the case of simulated damage presence as well as undamaged structure condition. All repetitions of measurements are included for the purpose of comparison. For the attached network of PZT sensors, all of the sensing paths are considered (Figure 15a), whereas for the embedded network the sensing path G-S3 is excluded from the plot (Figure 15b), due to abovementioned divergence of the results obtained for it.

Remarkably, DIs obtained for different sensing paths under simulated damage condition are located in similar domains of the complex plane. This can be observed both for the attached and the embedded sensors. The material used for the experiment is anisotropic, so the PZT sensors also exhibit some anisotropies when interacting with elastic waves [5]. However, there is no significant dependence of the results (Figure 15) on the direction of sensing path or orientation between the actuator and the receiver (Figure 5). Also, the length of sensing path does not influence the DIs’ distribution. This property is of particular importance for damage classification possibility based on the proposed DIs. Since the DIs are located at the same region of the complex plane for comparable measurement conditions, it is possible to apply statistical methods for damage classification, e.g., nearest neighbor algorithm, for damage classification [44].

**Figure 16:**
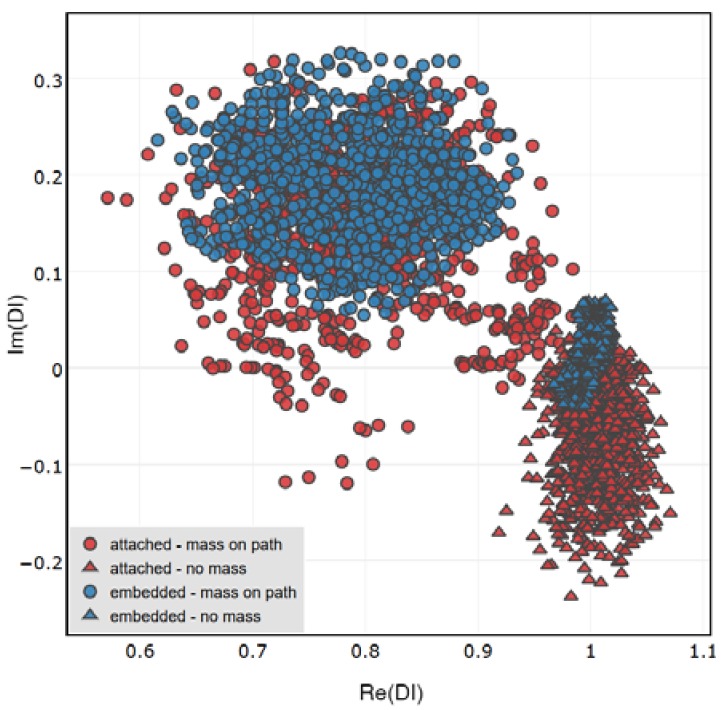
Comparison of DIs obtained for the embedded and surface attached PZT networks under certain measurement conditions.

In Figure 16 the performance of the attached and embedded sensors in damage indication is compared. For both networks DIs obtained for sensing paths G-S1 and G-S2 were included. The bias of DIs distribution from the point 1 + *i*0 is similar for attached and embedded sensors, the difference between them is revealed in the separation of data corresponding to undamaged and damaged structure.

**Figure 17:**
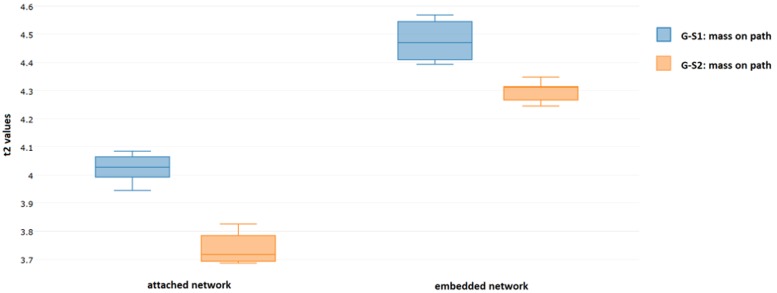
Values of T2 statistics obtained for both PZT networks.

The separation of data can be quantified by using Hotelling’s T-squared distribution (T2) [45]. It is used for statistical testing of difference between means of multivariate random samples. Higher values of T2 distribution obtained for two groups of multivariate data indicates more significant separation of the two groups. In Figure 17 boxplots of values of T2 distribution obtained for DIs calculated for repetition of measurements are presented. The values of T2 were obtained as:

(10) $$t^{2}=\frac{n_{d}n_{u}}{\left(n_{d}+n_{u}\right)}{\left({\bar{DI}}_{d}-{\bar{DI}}_{u}\right)}^{T}\Sigma^{-1}\left({\bar{DI}}_{d}-{\bar{DI}}_{u}\right),$$

where:

(11) $$\Sigma =\frac{(n_{d}-1)\Sigma_{d}+(n_{u}-1)\Sigma_{u}}{n_{d}+n_{u}-2}$$

and $n_{i},\;\;{\bar{DI}}_{i},\;\;\Sigma_{i}$ for i=d,u denotes respectively: the number of data points, two dimensional mean of DIs and sample covariance matrix obtained for damaged and undamaged state of the structure.

The separation between data corresponding to damaged and undamaged state of the structure is higher for the embedded sensors. Therefore, for this technology involving PZT sensor integration with the structure, it might be possible to achieve higher precision of damage classification models. The space spanned by the proposed DIs in such case might be able to accommodate more well-separated domains, corresponding to different types of damage. This however, comes at the price of more complicated process of components manufacturing, in particular taking into account possible adverse effects (Figure 13b) due to improper configuration of wires connected to the embedded sensors Figure 14.

## 5. Conclusions

In the paper a method for structure monitoring by PZT networks was proposed and its basic properties has been studied. The proposed method revealed stability of damage indication, irrespective of the relative orientation of PZT transducers, geometrical properties of the PZT network or anisotropies of the tested material. This property is of particular importance for possibility of damage classification. For structure-embedded sensors the indication of damage is more significant compared to that of surface-attached sensors. However, proper configuration of wires connected to embedded sensors needs to be assured, which is an additional manufacturing difficulty in this technology of sensors integration with the structure.
